# Designing an Embedded Feature Selection Algorithm for a Drowsiness Detector Model Based on Electroencephalogram Data

**DOI:** 10.3390/s23041874

**Published:** 2023-02-07

**Authors:** Blanka Bencsik, István Reményi, Márton Szemenyei, János Botzheim

**Affiliations:** 1 Department of Control Engineering and Information Technology, Budapest University of Technology and Economics, Magyar Tudósok Körútja 2, 1117 Budapest, Hungary; 2 Department of Artificial Intelligence, Faculty of Informatics, ELTE Eötvös Loránd University, Pázmány Péter Sétány 1/A, 1117 Budapest, Hungary

**Keywords:** feature selection, drivers’ drowsiness detection, EEG signals, driving automation

## Abstract

Driver fatigue reduces the safety of traditional driving and limits the widespread adoption of self-driving cars; hence, the monitoring and early detection of drivers’ drowsiness plays a key role in driving automation. When representing the drowsiness indicators as large feature vectors, fitting a machine learning model to the problem becomes challenging, and the problem’s perspicuity decreases, making dimensionality reduction crucial in practice. For this reason, we propose an embedded feature selection algorithm that can be later utilized as a building block in the system development of a neural network-based drowsiness detector. We have adopted a technique: a so-called Feature Prune Layer is placed in front of the first layer in the architecture; as a result, its weights change regarding the importance of the corresponding input features and are deleted iteratively until the desired number is reached. We test the algorithm on EEG data, as it is one of the best indicators of drowsiness based on the literature. The proposed FS algorithm is able to reduce the original feature set by 95% with only 1% degradation in precision, while the precision increases by 1.5% and 2.7% respectively when selecting the top 10% and top 20% of the initial features. Moreover, the proposed method outperforms the widely popular Principal Component Analysis and the Chi-squared test when reducing the original feature set by 95%: it achieves 24.3% and 3.2% higher precision respectively.

## 1. Introduction

Several factors might cause driver drowsiness, including sleep deprivation, physical exhaustion, medication side effects, and monotony. The last one is even more significant in the case of automated driving, where, due to the lack of active involvement, the driver is prone to become fatigued. At levels 2 and 3 on the driving automation scale defined by the Society of Automotive Engineers (SAE), the driver is out of the loop for prolonged periods, however, they are expected to take over the control in certain scenarios [1]. This might lead to severe consequences if the driver is not alert and fails to perform a critical action. Therefore, detecting drivers’ drowsiness not only increases the safety of manual driving, but it also facilitates the widespread adoption of automated driving. For this reason, the development of a reliable machine learning-based driver assistant drowsiness detector system is a currently active, widely studied research topic.

Nevertheless, various methods have been proposed for the identification of drivers’ drowsiness using different indicators, such as subjective self-assessment, expert assessment, reaction time measurements, the percentage of eyelid closure over the pupils (PERCLOS) and other physiological signals, like electroencephalograms (EEG) describing brain function, electrooculograms (EOG) representing eye movements, electrocardiogram (ECG) representing heart waves, breathing, etc. [2]. Unfortunately, in practice, all these indicators can be described as large feature vectors at every timestamp, which enlarges the input data’s dimensionality significantly, also it is likely to contain redundancy. When dealing with machine learning problems, high dimensional input raises various issues, for example, it increases the space and computational complexity, makes the clustering of similar features challenging, and increases the risk of overfitting the machine learning model [3]. Moreover, it decreases the perspicuity and the testability of the given system. Many dimensionality reduction methods exist to overcome these issues, originated to the fact that the optimal feature subset selection for any given estimator is already proven to be an NP-hard problem [4].

### 1.1. Objectives and Contribution

- **Motivation.** Our work aims to find a high dimensional input’s smallest available feature subset that contribute the most to the classification of the driver drowsiness without causing relevant performance drop in the selected metrics.
- **Contribution.** Implementation-wise, the proposed feature selection method was inspired by a state-of-the-art (SOTA) embedded feature selection algorithm that exploits the neural network updates’ working principle for selecting the features with the highest predictive power, namely that its weights change depending on the actual relevance of the input. We performed its domain adaptation, namely, we have modified the algorithm to be able to handle extremely redundant high dimensional input data.
  Another part of the work is the definition of an adequate data set that can model the driver drowsiness well enough. EEG-based features have been proven to be one of the best indicators of drowsiness as a detector model is able to provide accurate predictions when trained with EEG only without other sources of information [5]. Due to the characteristics of the sensor used to measure it, a large number of features can be extracted from the raw recordings, therefore, we extended the selected benchmark set within a preprocessing step.
  We also provided the evaluation of the proposed method as a comparison between different kind of FS methods, a reproducibility test and insights about its behavior.
- **Benefits.** The proposed method ensures to reveal complex, non-linear relations between the features during the training of the detector network and maximizes the amount of drowsiness-related information extracted from a set of EEG features extracted from the raw signal. As a result, we were able to reduce the number of features by 95% with a minor deterioration in the model’s precision and to produce a more accurate prediction when deleting 80% and 90% of the initial features. Furthermore, the efficiency of the proposed method is also proven by the fact that it outperforms the popular Principal Component Analysis (PCA) and the Chi-squared test feature selection algorithms.

### 1.2. Paper Organization

In this paper, the reader can first find a literature overview (Section 2) about the basics of working with EEG signals and how they can be utilized for drowsiness detection, the high dimensionality-related problems in the field of machine learning and basics of feature selection methods. This is followed by a brief introduction to the SOTA embedded feature selection method that primarily inspired our work. After that, we define the problem to be solved and briefly introduce the proposed method’s architecture in Section 3. In Section 4, we can find a detailed description of the goal-directed modification of the initial SOTA method to make it suitable for solving the defined problem. In Section 5, we present the achieved results and evaluate them in terms of the performance of the models trained on the original feature set and reduced feature sets produced by traditional feature selection algorithms and by our method. In  Section 6, we discuss the proposed method’s advantages and disadvantages and the credibility of the selected features based on the literature. Finally, in Section 7 conclusions are drawn.

## 2. Preliminaries

### 2.1. Driver Drowsiness Detection

The field of driver drowsiness detection has been actively studied in the past decades, and several solutions have been proposed. Drowsiness detection methods are commonly grouped into the following categories based on the source of the data used for the detection:

1. behavior-based,
2. vehicle-based,
3. physiological signal-based,
4. hybrid methods.

The non-invasive behavior-based methods measure fatigue levels using parameters like eye closure ratio, eye blinking, head position, facial expressions, and yawning. From these parameters, behavioral features are extracted with the help of cameras and computer vision techniques. One of the most frequently used metrics in this category is the Percentage of Eye Closures (PERCLOS), which is the ratio of eye closures over a period. Vehicle-based methods aim to detect fatigue from the different states of the vehicle, such as lane-changing patterns, speed variability, steering wheel angle, etc. To collect these types of data, various sensors are required on the vehicle’s different parts. Finally, physiological signal-based approaches detect drowsiness based on the subjects’ physiological condition, such as heart rate, brain changes, respiration, body temperature, etc. To measure these invasive biological parameters electrodes need to be placed on the subjects’ body [6].

The classification method determines the resolution of the detection: threshold-based and binary classification methods distinguish between drowsy and alert stages, in contrast multi-class classification methods can predict several levels of fatigue. Multi-class classifiers are more suitable for estimating the severity of drowsiness, since they can detect the drowsiness in its early stage and provide early warning. Unlike the aforementioned methods that predict discrete labels, regression methods can estimate continuous variables. The most widely used decision-making models are radial basis functions (RBF), support vector machines (SVM), artificial neural networks (ANN), fuzzy inference systems (FIS), linear discriminant analysis (LDA), receiver support vector regression (SVR), multiple linear regression (MLR), self-organizing neural fuzzy inference networks (SONFIN), etc. [2].

In the case of supervised machine learning-based decision-making models, the ground truth is used to label the training data; to determine the true drowsiness value of the events. Having a reliable ground truth is crucial as its precision directly implies the exact characteristics of the decision-making model. Ground truth can be obtained by subjects’ self-assessment, expert rating, reaction time, and physiological signals [2]. In many studies, EEG has been reported to be the most reliable indicator of drowsiness, as it directly describes the drivers’ physical state [5,7]. However, its main drawback is that it requires sensors to be attached to the driver’s body, which may obstruct them. In addition, EEG signals might vary based on the subjects’ age, gender, physical state, etc. [8].

Several aspects must be considered when developing a drowsiness detector system. Usually, the data acquisition is cumbersome and expensive, as it requires either an environment simulator or a vehicle equipped with all the necessary expensive sensors. In addition, most measures used for producing ground truth data are highly subject-dependent. These factors make the development of an effective, reliable driver drowsiness detector extremely challenging.

### 2.2. EEG Features

#### 2.2.1. Measuring EEG Signals

Electroencephalography measures the electrical activities of different brain regions using surface electrodes placed on the scalp. EEG is a graphic display of potential differences between two sites of the brain recorded over time [9]. EEG can be used to diagnose several medical conditions, such as epilepsy, Parkinson’s Disease, autism, anxiety, sleep disorders, insomnia, and many more. Moreover, different research fields have also utilized it, namely brain-computer interfaces, biometrics, neuroscience and clinical applications, and neuromarketing [10].

**Figure 1:**
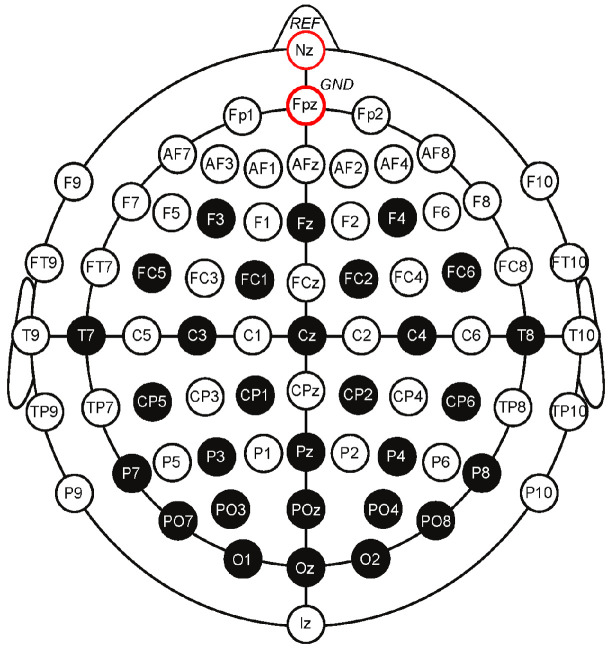
The 10–20 electrode system of the International Federation [12].

The International Federation of Clinical Neurophysiology standardized the electrode placement into the 10–20 system. This system requires the use of at least 21 electrodes and enables the measurements to be proportional to the size and shape of the skull, provides adequate coverage of the entire head, and expresses the electrode designations in terms of brain areas. The designations consist of a letter that refers to the region of the brain (F: frontal, C: central, T: temporal, P: posterior, and O: occipital) and of a number that differentiates between left and right homologous regions—odd numbers indicate the left, even numbers indicate the right hemisphere, while “z” designation refers to the midline—in such way, that lower numbers reflect positions closer to the midline (Figure 1) [11].

#### 2.2.2. EEG Feature Extraction

**Figure 2:**
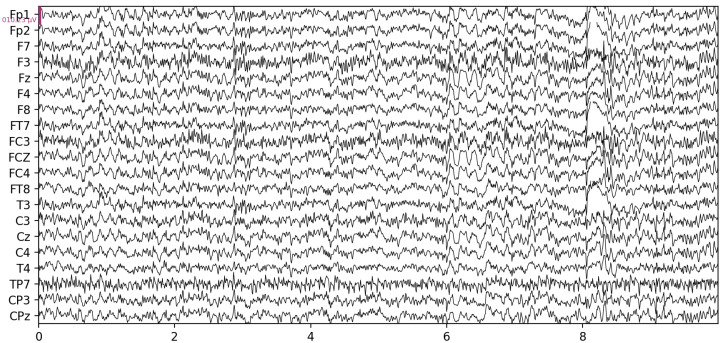
Raw EEG signals recorded on various electrodes.

A wide range of features can be extracted from raw EEG data (Figure 2) describing its characteristics which are used in different applications, however, in this paper, we only focus on features relevant to drowsiness detection. Based on [13], these can be categorized into the following groups:

1. time-domain (mean, median, variance, skewness, number of zero-crossing, etc.),
2. frequency-domain,
3. nonlinear,
4. entropies,
5. undirected spt.,
6. directed spt.,
7. complex networks.

Among these categories, the FFT-based features are the most commonly used in drivers’ drowsiness detector systems, most popularly with a 1 min time window for extracting the features [2]. The Power Spectral Density (PSD) of the signal plays a vital role in calculating the frequency-domain features. It can be obtained with the Fast Fourier Transform algorithm (FFT) [14]), Welch’s method [15], or the Thompson multitaper method.

Besides the widely favored Fourier Transform, the signal can be transformed from the time domain to the frequency domain using wavelet decomposition [16] or matching pursuit decomposition [17] as well. While Fourier Transform decomposes the signal into sinusoids, in the case of wavelet decomposition, the decomposition is done by an underlying mother wavelet function. According to [13], the most frequently used frequency-domain features in all fields of EEG analysis are the relative powers of the most commonly used frequency bands, namely: delta (δ, 0.5–4 Hz), theta (θ, 4–8 Hz), alpha (α, 8–12 Hz), beta (β, 12–30 Hz), and gamma (γ, >30 Hz). However, different ratios between these bands also appear in EEG signal analysis: $\frac{\theta +\alpha }{\beta },\frac{\alpha }{\beta },\frac{\theta +\alpha }{\alpha +\beta },\frac{\theta }{\beta },\frac{\theta }{\theta +\alpha },\frac{\alpha }{\theta +\alpha },\frac{\theta +\alpha }{\theta +\beta }$ [18,19].

### 2.3. Feature Selection (FS)

#### 2.3.1. Dimensionality Reduction in Machine Learning

In today’s digital era, a tremendous amount of data is generated every second with high-dimensional features, which are ubiquitous in various data science fields. When applying data mining and machine learning models on high dimensional data, the Curse of Dimensionality (CoD) phenomenon is likely to occur: the volume of the space increases together with the dimensionality, causing the data to become sparse [20]—this usually means the features having zero values. A model trained with sparse data is prone to learning the noise, it cannot generalize well, which leads to overfitting and performance degradation on unseen data [21]. Besides, high dimensional data enhances the computational burden and decreases the perspicuity and the testability of the given problem.

Many dimensionality reduction techniques have been introduced to alleviate the aforementioned obstacles. During the dimensionality reduction process, the the redundant and irrelevant features are omitted, yielding a more compact, more easily interpretable representation of the target concept with the most relevant features [22]. Dimensionality reduction is commonly categorized into two main groups: feature extraction (FE) and feature selection (FS). Feature extraction compresses the high-dimensional feature set into a smaller one by constructing a new, lower-dimensional feature space, usually by applying linear or nonlinear projection of the original set. It is preferred in applications where only the raw data is available, which is not interpretable for a learning algorithm. However, in this case, the problem of further analysis arises, as we cannot retain the physical meaning of the new features. On the other hand, FS means selecting a subset of relevant features from the initial set, keeping the physical meanings of the original features [21].

#### 2.3.2. FS Categories Based on Selection Strategy

**Figure 3:**
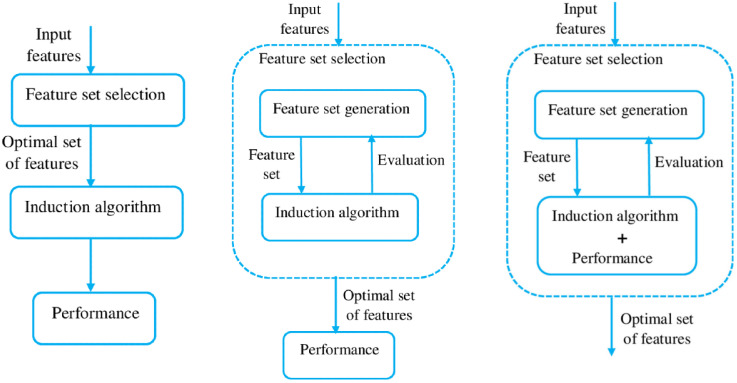
Flowchart of the FS process in different FS categories: Filter, Wrapper, Embedded [25].

FS is one of the most used dimensionality reduction methods. Its general working principle consists of four main steps: generation of a feature subset, evaluation of the feature subset, checking the termination condition, and result validation [23]. FS methods can be categorized based on different perspectives. In terms of the availability of the labels in the training data set, they can be divided into supervised (labels are available), unsupervised (labels are not available), and semi-supervised methods [21]. Aligned with the original problem statement, a supervised solution was preferred in this study. Another categorization type relies on the selection strategy and distinguishes three main methods: filter, wrapper, embedded (Figure 3) [24].

##### Filter Methods

Filter methods utilize the data’s intrinsic properties to assess feature importance. They calculate a score for each feature using different evaluation criteria that can be univariate (examining each feature individually) and multivariate (multiple features are examined together in a batch). The features are then ranked according to these scores, and a specified number of them with the lowest scores are filtered out, resulting in the most predictive subset of features. In the case of filter methods, the selection is performed before the model training, therefore, the FS is considered a pre-processing step. Among its most significant character traits, its independence from any learning algorithm should be mentioned, which makes filter methods usually faster than others, but raises the risk that the selected features may not be optimal for the given algorithm [21,24].

Several evaluation criteria exist for separating the features which approach the problem from different perspectives. The first option is to examine feature discriminative ability and select features so that within-class distance [26] is as small as possible while between-class distance [26] is as large as possible [27], meaning that features that strongly represent the given class and differ the most from features in other classes are selected into the subset. Some popular algorithms based on the aforementioned principle are the Fisher Score [28] and the Linear Discriminant Feature Selection [29] algorithms. Another idea is to exploit correlation measures, either to remove redundant features that can be applied in the case of unsupervised learning as well, or to select the most similar—highly correlating—features to the target variable if labels are provided. For the first scenario, PCA is a widely used method. For the latter scenario, various statistical measures can be used, including Pearson’s correlation coefficient (linear), ANOVA correlation coefficient (linear), Sperman’s rank coefficient (nonlinear), Kendall’s rank coefficient (nonlinear), the Chi-squared test and mutual information analysis. Their applicability for a given problem depends on the data variable types.

##### Wrapper Methods

In contrast to the filter methods, in the case of wrapper FS methods, the learning algorithm has to be defined, wrappers exploit their black-box nature to score subsets of features according to their importance and predictive power. Wrappers work iteratively, repeating the following steps until a stopping criterion is satisfied: they generate a subset from the initial features, which are then evaluated with the help of the predefined learning algorithm [21]. The stopping criterion is usually defined as the combination of the desired number of selected features and the highest possible learning performance achieved when training the model with this subset.

Besides the learning algorithm and the stopping criteria, the space search strategy also has to be selected. Sequential search methods (also called hill-climbing or steepest ascent) are search strategies that use greedy techniques to examine features sequentially. They either start from the initial set of features and eliminate them one by one (sequential backward selection (SBS)) or start with an empty set and add features one by one (sequential forward selection (SFS)). One shortcoming of these methods is that they can only guarantee local optimality. Genetic algorithms add some randomness to the search procedure, hence helping to overcome the local optimum problem [24,30,31,32]. Other feature subset selection algorithms are the best-first search, branch-and-bound search, etc. [30,33].

Unfortunately, it is perceptible that in terms of speed, wrapper methods are not efficient, due to the huge search space—$2^{N}$ where *N* is the number of features [24], which is even more problematic when dealing with very large sets of features. While some criticize this property of theirs and blame it for wrappers’ rare application in practice [21], others claim that choosing an efficient search strategy can alleviate this obstacle [34].

##### Embedded Methods

The embedded FS method is a trade-off between the filter method’s high speed but low accuracy and the wrapper method’s high accuracy, but expensive computational requirements. According to its descriptive name, in the case of embedded methods, the FS is integrated into the selected machine learning model’s training procedure; the best feature subset is produced during the training of the chosen learning algorithm. Therefore, the performance of the model highly depends on the selected features. It has the merits of interacting with the model, but due to the lack of iterative feature subset evaluation, it is significantly more efficient than wrapper methods [21]. Similar to the wrappers, embedded methods are also not confined to supervised FS and can be applied for unsupervised feature selection [24].

The most widely used embedded methods are the regularization methods, which aim to minimize fitting errors in order to fit the model to the feature set. To do so, they force feature coefficients to be as small as possible simultaneously [21]. Some popular examples of the regularization approach are the LASSO, RIDGE, and Elastic Nets. An embedded FS can also be done by any kind of tree-based algorithm, such as Decision Tree, RandomForest, ExtraTree, etc. [35].

### 2.4. FS Methods Used in This Paper

In this subsection, we are going to briefly summarize the widely popular conventional FS methods that are used as baselines in this paper to evaluate the performance of the proposed method. After that, the SOTA FS method that inspired our work in the first place is introduced in detail.

#### 2.4.1. Conventional FS Methods

- **Principal Component Analysis (PCA).** PCA relies on linear algebra techniques, and is widely used due to its easy application and non-parametric property. It projects the original, high-dimensional data into new dimensions to re-express it and explore hidden qualities. In a mathematical sense, the goal is to find the most meaningful basis by performing basis change transformations [36]. The summarized computation complexity of PCA is O($d^{2}$n + $d^{3}$), this comes from the O($d^{2}$n) covariance matrix time complexity and O($d^{3}$) eigen-value decomposition; where d stands for the number of features, n is the number of samples in the dataset.
- **Univariate FS.** The two test methods used in this paper are the Chi-Squared Test [37] and the Mutual Information (MI) Analysis [38]. These filter methods select predefined number of most contributing features based on $\chi^{2}$ test and entropy based dependency measurement, respectively. They both serve as an additional step to a given estimator algorithm, bringing additional training cost of O($n^{2}$) as a naive implementation and O(n∗log(n)) as a purpose optimized version [39,40].
- **Recursive Feature Elimination (RFE).** As a wrapper FS method, it requires an input scoring estimator to assign weights to the input features. Based on these values, the most relevant features can be pruned recursively [41]. Currently, we use a decision tree [42] for our comparison, which means O(n∗log(n)∗d∗log(d)) train time complexity.

#### 2.4.2. Stepwise Weight Pruning Algorithm (SWPA)

**Figure 4:**
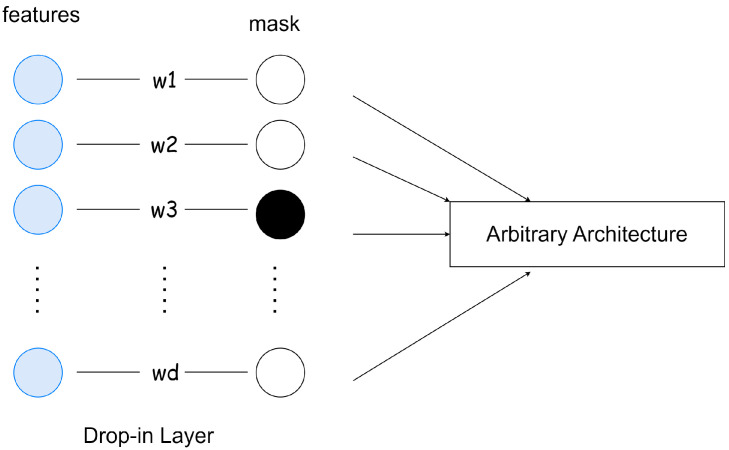
Modified network with our drop-in layer interpretation.

SWPA is a novel embedded FS method proposed by [43]. Its main idea is to incorporate a so-called drop-in layer into a neural network architecture and prune its weights iteratively until the most important ones are left (Figure 4). Weight pruning refers to the process of removing parameters from an existing, accurate network. The method exploits the neural network updates’ working principle, namely that its weights change depending on the actual relevance of the input:

(1) $$weight_{i+1}=weight_{i}+learning\_rate\cdot \frac{\partial error}{\partial weight_{i}}$$

If the drop-in layer ($W\in R^{1\times d}$ where *d* is the number of input elements) is the first layer in the network, and its weights are initialized to ones, the output of this layer $O=\{w_{1}x_{1},\cdots ,w_{d}x_{d}\}$ will be the multiplication of the corresponding input elements. Hence, if we set a weight $w_{i}$ to 0 in the drop-in layer, then that directly means that we removed input element $x_{i}$. Algorithm 1 summarizes the method’s working principle. By applying this concept as an additional layer, in which only an element-wise multiplication is present, just O(d) time complexity will be added to the original training cost of a selected neural network architecture; where d is the number of features. The SWPA has been tested on HAR, ISOLET, and MNIST data sets.
**Algorithm 1** Original Stepwise Weight Pruning Algorithm (SWPA) [43]**Input:** training data $X\in R^{n\times d}$, training labels *Y*, base network $f_{\theta }\left(.\right)$, Drop-in Layer *W*, Step Counter $n\in Z_{⩾1}$. Selection factorf∈[0,1]**for** count in 1,⋯,n+1 **do**    $O\leftarrow \{w_{1}x_{1},\cdots ,w_{d}x_{d}\}$    **if** count > 1 **then**        *k*$\leftarrow \frac{(1-f)\cdot d}{n}$        Sort the weights *W* of the Drop-in Layer based on their absolute value.        Set the least *k* of them to 0.    Train the base network on *O*Take the features corresponding to the top *f* fraction of the weights in *W* based on their absolute value and train them on the base network.

For the experiments, they use a 3-layer feedforward neural network with a reduction factor of 2, which is trained for 20,000 epochs if there is no performance degradation on any continuous set of 2000 epochs. The most important variables in the SWPA are:

1. the Step Counter (*n*): the features that have to be deleted to reach the desired number are removed in equal-sized groups in *n* steps.
2. the Selection Factor (*f*), which defines what percent of the initial feature number should be selected into the final subset.

According to the paper, these variables are set to the following values: n=4, f=0.1. To evaluate the achieved results, besides the random assignment, they use the Permutation Feature Importance (PFI) as a baseline with the number of random permutations of 10 which is an importance attribution technique commonly used for random forests. SWPA outperforms both the random assignment and the PFI on all datasets when the 10% of the original number of features is selected: for example, on the MNIST dataset it yielded a 0.941 accuracy, while using PFI the achieved accuracy was 0.893, and with random selection 0.714. With these results, SWPA has proved itself to be a simple, yet efficient embedded FS method, which is easy to apply in various tasks as the drop-in layer can be incorporated into any neural network architecture [43].

In Table 1 a comparison of the above mentioned FS methods can be found.

## 3. Problem Statement

Our work aims to design and implement an FS algorithm with feature set purification capabilities that can be later utilized during developing a driver’s drowsiness detector. According to the outstanding results achieved by SWPA introduced in Section 2.4.2, it’s easy applicability, and embedded property, we have found it to be a satisfactory choice for the basis of the designed FS method. However, the paper stays vague about the implementation of the drop-in layer. Although the description states clearly that the feature scoring depends on the weights in the drop-in layer which is the first layer of the used neural network architecture—therefore its weights change according to the importance of the input elements—, by observing Algorithm 1, the drop-in layer seems to be left out of the parameter update, as they always retrain the base network on its output [43].

**Figure 5:**
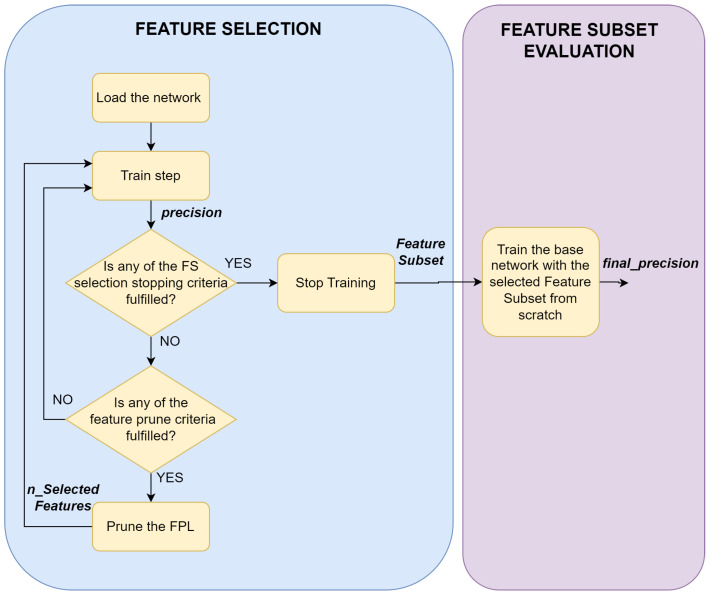
Flowchart of the proposed FS algorithm.

For this reason, we rethink the idea proposed by [43], and completed it with additional properties to make it better suitable for the introduced problem. The flowchart of the final algorithm can be seen in Figure 5. This solution also strives to exploit the neural network updates’ working principle, hence, we implement a layer similar to the drop-in layer, called Feature Prune Layer (FPL), which has the same size as the number of input features and is in a one-to-one relationship with them. The network is then trained until any FS stopping criteria are fulfilled. The FPL remains part of the network for the whole training process, and a pruning step is performed on it if any of the feature prune criteria is fulfilled. The pruning consists of removing the dinamically calculated number of weights from the FPL with the lowest magnitudes. The FS part is followed by the feature subset evaluation when the base network—without the FPL—is retrained from scratch with the selected feature subset. These two main parts are considered the proposed FS algorithm, and the performance is deduced from the precision achieved in the feature subset evaluation part.

Summarily, according to the literature, the proposed algorithm’s task is to select the defined number of best predictive features from a set of EEG-based features that are feasible for driver drowsiness detection. To find the best setup of the algorithm, several tests have been performed examining the impact of the different hyperparameters. In the following section, we detail the aforementioned goal-directed modification of the SWPA, the feature pruning and FS stopping criteria, the hyperparameters in the model, and the motive for their selection.

## 4. Materials and Methods

### 4.1. Development Environment

We implement and run the codes in a Python 3.8.10 environment on an Ubuntu 20.04.5 LTS operating system powered by an Intel(R) Xeon(R) Gold 6248R CPU and an NVIDIA T4 Tensor Core graphics card. The pre-processing of the EEG signals is achieved with the help of the MNE-Python package [45] and the neural network model is built using the PyTorch framework [46].

### 4.2. Used Metrics during the Development

To introduce the design and planning process of the proposed solution, it is crucial to keep the final goal in mind, which includes the awareness of the desired outcome measured with the chosen metrics. Intending to make the following subsections easily readable, we introduce the two main metrics used during the design and development of the FS algorithm. For keeping track the performance changes on unseen data, from the previously defined 70% train set a randomly selected 10% was nominated as validation set.

- **Precision (Macro Averages) [%]**: This metric is defined as the precision calculated separately for each individual class, averaged over all the classes. Ideally, this value is determined independently for the training set during the training of the classifier and for the validation set, which is carried out after each specified number of iterations (epochs).
- **Pseudo-Overfitting [%]**: The difference between the train and validation precision refers to the generalization ability of the classifier. If the validation precision is lower than the train precision, it implies the so-called overfitting phenomenon: the classifier is unable to perform well on unseen data. Here, we define the pseudo-overfitting metric as the signed difference between the train and validation precision values. Therefore, the aim is to achieve as small pseudo-overfitting value as possible.

### 4.3. Data Preparation

#### 4.3.1. Multi-Channel EEG Recordings Dataset

**Figure 6:**
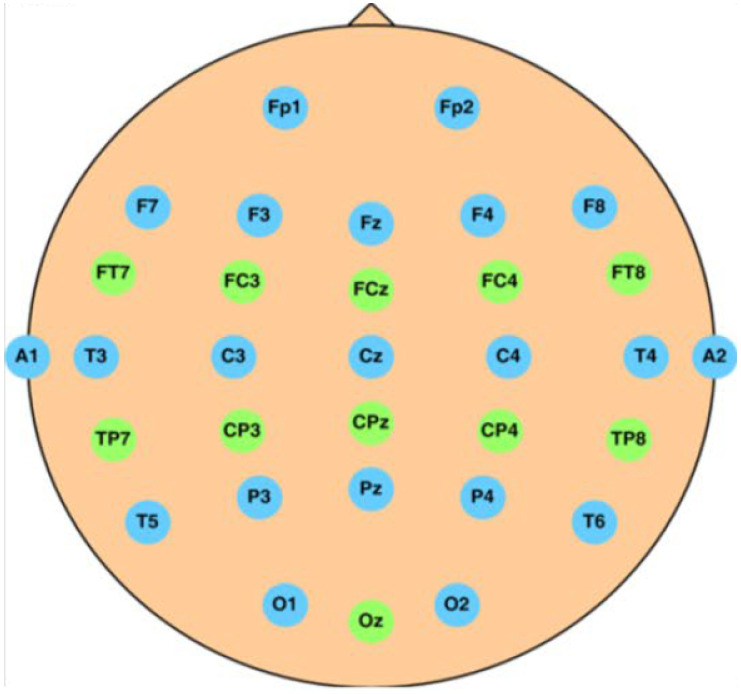
The layout of the electrodes in the EEG cap used for the experiments in [48].

In this study, the [47] public data set (processed) is used, which is a processed version of [48] (original). The original data set contains multi-channel EEG recordings recorded during a sustained-attention driving task with the help of 27 subjects (aged between 22–28). During a 90-min experiment conducted in a VR driving environment with a dynamic driving simulator, the subjects were asked to keep the car in the center of the lane and respond quickly to the randomly introduced lane-departure events. These perturbations made the car drift to the left or the right side of the lane (deviation onset). For obtaining the drowsiness level of the driver, in addition to the deviation onset, response onset (the subject steering the wheel in case of a departure event) and response offset (the car arriving back to its original position) occurring times have been recorded. These indicators of the drivers’ promptness are instantaneous measures of the drowsiness level that can be calculated using the method described in [49]. The EEG signals were collected with the help of a wired EEG cap (Figure 6) with 32 Ag/AgCl electrodes (of which two were used as reference) based on a modified International 10–20 system [48].

The authors of paper [50] have produced the processed, balanced version of the original, pre-processed data set where the EEG data were digitalized at 500 Hz, a 1-Hz high-pass and 50-Hz low-pass filter was applied to it, followed by artifact rejection. They down-sample the EEG signals to 128 Hz, then extract equal-long, 3-s samples. Each sample was labeled with a 2-state drowsiness level—drowsy or alert—using the aforementioned [49] method. The authors have devoted special effort to creating a compact, balanced data set, containing the most representative samples from different subjects, by carrying out the following steps:

1. They have discarded sessions where the number of samples from either class is less than 50.
2. In case of multiple sessions belonging to the same subject, they have chosen the one with the most balanced class distribution.
3. From each session, they selected alert samples with the shortest- and drowsy samples with the longest response time.

Steps 1 and 3 ensure the the classes are balanced, while step 2 results in balanced data from different subjects, hence, it is not likely that the classifier will be prone to favor the prediction of a specific subject. The final data set contains 2022 3-s long, pre-processed EEG samples collected from 11 different subjects [50].

#### 4.3.2. EEG Feature Extraction

The chosen data set introduced in Section 4.3.1 contains 3-s long time-domain EEG signals, referred to as segments. In order to convert these signals into an interpretable format for any classification algorithm, we have to extract features that comprehensively describe the data set. Therefore, for every segment, the commonly used frequency-domain EEG features introduced in Section 2.2.2 are determined:

$$\alpha \text{-}\mathrm{PSD},\;\beta \text{-}\mathrm{PSD},\;\theta \text{-}\mathrm{PSD},\;\frac{\theta +\alpha }{\beta },\frac{\alpha }{\beta },\frac{\theta +\alpha }{\alpha +\beta },\frac{\theta }{\beta },\frac{\theta }{\theta +\alpha },\frac{\alpha }{\theta +\alpha },\frac{\theta +\alpha }{\theta +\beta }$$

The Power Spectral Density (PSD) is calculated with the help of Welch’s method [51], using a window size of 3 s. These aforementioned features are obtained from the signals measured individually on every electrode found on the EEG cap used to record the signals (Figure 6). In addition, the calculated values are averaged over the frontal, the temporal, and all the electrodes, as—according to the literature—some EEG frequency bands are more active on the frontal or the temporal part of the brain. Namely, these electrode positions are [48]:

Fp1, Fp2, F7, F3, Fz, F4, F8, FT7, FC3, FCZ, FC4, FT8, T3, C3, Cz, C4, T4, TP7, CP3, CPz, CP4, TP8, A1, T5, P3, PZ, P4, T6, A2, O1, Oz, O2, frontal, temporal, all

**Figure 7:**
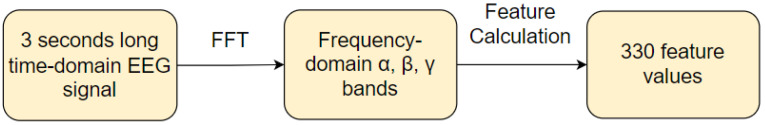
The process of feature extraction from time-domain EEG segments.

**Figure 8:**
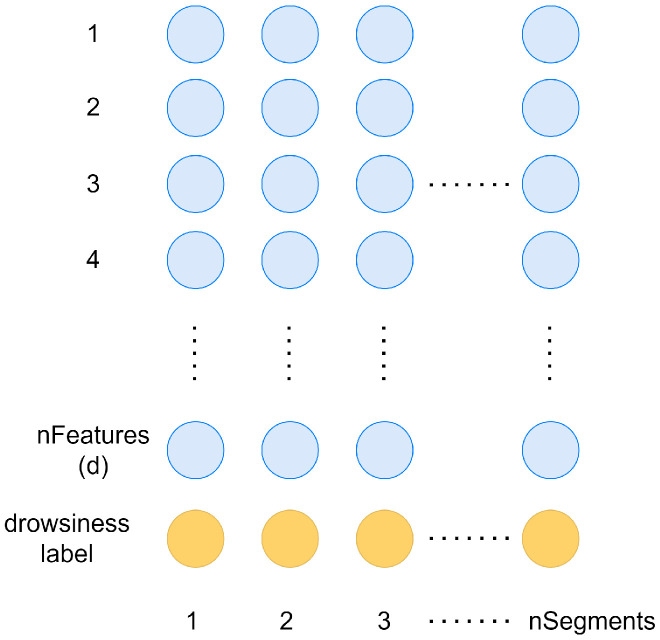
The structure of the generated EEG data set.

These calculations (Figure 7) have resulted a 330-element feature vector for each 3 s long EEG segment (Figure 8). This feature set serves as the input for the designed FS algorithm, which aims to select the desired number with the highest predictive power. For the development phase, the data set has been split into train and test sets in a 70–30% ratio while ensuring that the labels stay balanced by not letting the difference between the number of drowsy and alert labels be greater than 20.

### 4.4. Development of the FS Method

#### 4.4.1. Iterative Feature Pruning

Similarly to the SWPA (Section 2.4.2), the feature scoring method of the proposed embedded FS algorithm also relies on the neural network updates’ working principle. An FPL is attached to the front of the classifier network (base network), which has the same size as the number of input features and is in a one-to-one relationship with them. Consequently, during the training, its weights change according to the importance of the input features, hence, deleting a weight from the FPL means the removal of the corresponding feature from the original feature set.

**Figure 9:**
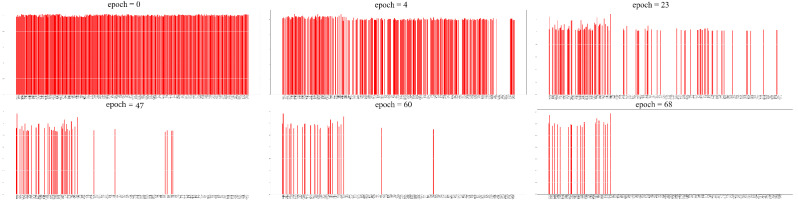
A few steps of the iterative feature pruning process. The red sticks represent the still remaining features and the vertical axis shows the corresponding scores for each feature. As we move forward with the training, the scores change according to the feature’s importance. At a given pruning step, the $N_{deleted\_weights}$ number of features with the smallest magnitude are deleted, until only the desired number of features remain.

If a predefined feature pruning criterion (see Section 4.4.2) gets fulfilled, a subset of the remaining weights with the smallest magnitude in the FPL will be deleted. The number of deleted weights in a pruning step are defined as follows:

(2) $$N_{deleted\_weights}=\left\lfloor \frac{f\cdot d}{n}\right\rfloor$$

where *d* corresponds to the remaining number of features in the FPL, *n* is a counter of the pruning steps and f∈[0.1,0.4] is a constant that directly contributes to the number of removed weights in a given step. Choosing a higher *f* results in a coarser pruning strategy. Nevertheless, the use of *n* prompts that the further we move with the training the more gentle the weight pruning gets. The feature scoring and the iterative feature pruning process are demonstrated by Figure 9.

#### 4.4.2. Feature Pruning Criteria

A pruning step is performed on the FPL if any of the following feature pruning criteria is fulfilled:

1. The test precision (prec) reaches a predefined value (final_prec)
2. The predefined number of epochs is reached (max_epochs)
3. The pseudo-overfitting (psovft) reaches a predefined level (max_psovft)

Commonly, when a neural network model is pruned, its performance slightly drops and it needs a few iterations of training to regain its earlier precision. Depending on the coarseness of the weight pruning defined by Equation (2) and the given training phase, the degradation of the precision varies in the different scenarios. For example, if $N_{deleted\_weights}$ is a large number, a significant amount of the weights is going to be deleted from the FPL even in the first pruning step, which is likely to cause a heavier precision degradation than if it was pruned with a smaller $N_{deleted\_weights}$. In addition, the longer we train the network, the more confident it gets, meanwhile, the pruned amounts will decrease due to their inverse relationship with the pruning step counter. Because of this, it is not ideal to train the network for the same number of epochs between each feature pruning step, as it may need a dissimilar amount of iterations to regain its precision. We use the test precision to determine the appropriate moment of the next pruning step. According to the first feature pruning criterion, the next pruning step can be performed if the network’s precision reaches the predefined final_prec after the last reduction.

It is possible that the network will never be able to reach the desired final_prec after a certain point. In order to prevent the training from getting stuck in an infinite loop, according to the second criterion, a feature pruning step may also be carried out if the network has been trained for a predefined maximum number of epochs (max_epochs) since the previous one. Lastly, a pruning step also takes place if none of the aforementioned criteria is fulfilled, but the pseudo-overfitting reaches the max_psovft threshold. This is likely to happen if $N_{deleted\_weights}$ is too small, and the pruning is performed at a slower pace than the network’s regeneration ability. A summary of the selected values for the previously discussed thresholds can be found in Table 2.

#### 4.4.3. Algorithm Structure

The embedded nature of the proposed FS algorithm is due to the fact that the FS process is carried out during the training of the classifier network, while continuously performing the feature pruning introduced in the previous subsections. The training may be terminated if any of the following FS stopping criteria is fulfilled:

1. The desired number (des_feat_num) of features is left in the feature set, which is the ideal case.
2. The network was trained for a maximum number of epochs (max_epochs_final). This ensures that the training will not get stuck in an infinite loop if the desired number of features cannot be reached with the selected hyperparameters.

After the termination, the final subset of selected features is evaluated on the base classifier network—the same architecture but without the FPL. This is considered as the end of the FS process, and the final results are the ones achieved with this step: the performance of the base classifier with the selected feature subset (newset_prec, newset_psovft). The whole process of the FS is demonstrated by Algorithm 2.
**Algorithm 2** Proposed FS Algorithm**Input:** original EEG feature set [1 × *d*]
network← initialize▹ FPL to ones, rest randomlyn← 1▹ pruning step counterepochs← 0▹ epochs between two pruning stepsepochs_final← 0▹ all epochs during trainingprec←0,psovft←0**while** (*d* > des_feat_num) OR (epochs_final<max_epochs_final) **do**    **if** (prec≥final_prec) OR (epochs≥max_epochs) OR (psovft≥max_psovft) **then**▹ Perform pruning on the FPL        $N_{deleted\_weights}$$\leftarrow floor(\frac{f\cdot d}{n})$        *d*← delete the $N_{deleted\_weights}$ weights in the FPL with the smallest magnitude        epochs ← 0        n = n + 1    prec,epochs,epochs_final← train()newset_prec, newset_psovft← take the new *k*-sized feature subset and train the base network on it from scratch

### 4.5. Hyperparameter Selection

While reading the previous subsections, it is apparent that during the FS process, some of the defined thresholds were handled as variables. The changing of these variables strongly influences the outcome of the FS algorithm: the composition of the final feature subset. While we might have an assumption about how the changing of these variables individually affects the outcome, the problem gets more complex if we combine them. Moreover, due to the neural networks’ black box nature, they act as hyperparameters and it is impossible to define their value consistently. The proposed FS method has two hyperparameters:

1. ***f***∈[0.1,0.5] parameter which directly contributes to the number of removed weights in a given step. Choosing a higher *f* results in a coarser pruning strategy (Equation (2)).
2. ***final_prec***∈[0.6,0.95] which is on the feature pruning criteria (see Section 4.4.2).

In order to determine the best selection of these hyperparameters for the FS method, we have conducted several experiments testing their different values introduced by Algorithm 3.
**Algorithm 3** Experiments For Hyperparameter Selectionresults←[]**for** final_prec in 0.6, …, 0.95 **do**    **for** *f* in 0.1, …, 0.5 **do**        newset_prec← select the des_feat_num of number of features from the original set with final_prec and *f*        results.append( newset_prec, newset_psovft)find the best performance in results and take the corresponding hyperparameters

### 4.6. Feature Prune Layer Realization

**Figure 10:**
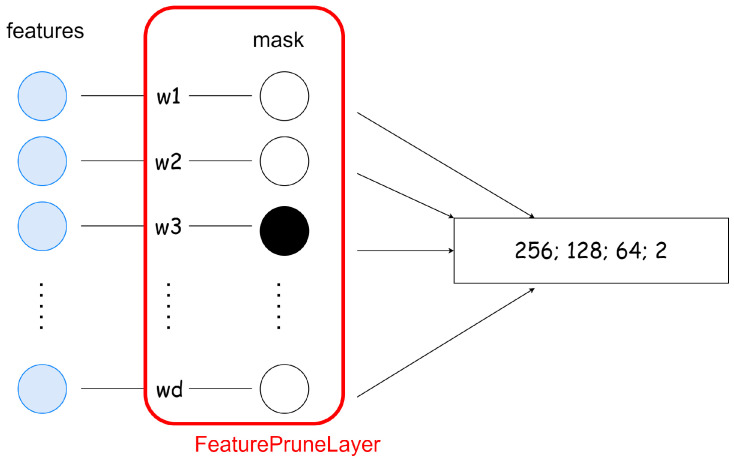
Architecture of the network used for FS.

In terms of the implementation, the FPL is realized the same way as a linear layer, but instead of matrix multiplication, it computes the Hadamard product between its weights and the input. When talking about neural network pruning, we can distinguish two main types based on the structure of the weight removal: structured and unstructured pruning [52]. In the case of structured pruning entire groups of weights are removed (like channels, filters, or layers), while unstructured pruning corresponds to deleting weights individually by setting their value to zero. For removing weights from the FPL unstructured pruning is used. Implementing unstructured pruning is difficult in practice, because during the parameter update, the zeroed weights in the networks’ layers also get updated regardless of their current value. To overcome this issue, instead of setting the weights in the layer to zero, we use a mask with which the weights of the FPL are multiplied in every forward pass. This way, in every pruning step, only the mask gets modified, therefore there is no need to detach the FPL from the computational graph, which avoids the decrease in the algorithm’s speed. The illustration of the FPL’s implementation can be seen in Figure 10.

### 4.7. Classifier Network

Similarly to the SWPA, we also use a small network with four hidden layers for the classification. In order to deduce the most beneficial size for these layers, we have trained multiple networks with different layer sizes on the whole feature set. Each training session ran for 70 epochs, using Adam optimizer, a batch size of 64, and an initial learning rate of 0.001. Every architecture has been tested four times and the average of the achieved results is demonstrated in Table 3. The do sign between the layers in the network architectures stands for the dropout layer which serves as a regularization technique by randomly zeroing out some elements of the input with probability *p* using samples from a Bernoulli distribution [53]. Here, we used probability p=0.5. The *prec* and *psovft* are defined the same way as described in Section 4.2. The ${}_{\mathrm{avg}}$ and ${}_{\mathrm{std}}$ extensions refer to the average and the standard deviation of the performed tests’ results, respectively.

Arch4 seems to provide the most stable training as the standard deviation of both metrics is the smallest in this case. Nevertheless, arch5 achieves the highest precision, and given that the rest of the metrics do not vary significantly between the different architectures, the 256; 128; 64; 2 network architecture is chosen to be used for the further experiments.

## 5. Results

### 5.1. Reducing the Feature Subset

The aim of the experiments introduced in this subsection is to find out to what extent it is possible to reduce the feature set without a major degradation in the classification performance. The top 5%, 10%, and 20% of the original feature set are examined. For finding the desired number of features, the proposed FS method is tested with all the possible hyperparameter settings described in Section 4.5. As *f* varies in the range of [0.1, 0.4] with a step size of 0.1 and final_prec varies in [0.6, 0.95] with a step size of 0.05, all their variations resulted in 32 test cases for each feature subset size. Each test case resulted in a feature subset, with which the base network was trained 4 times, and the achieved results have been averaged. Table 4 summarizes these results: the three best performing test cases are shown for each feature subset, while Table 5 shows the case when the classifier is trained on the original feature subset.

The results indicate that it is possible to reduce the original number of features by 95% without significant performance degradation. In the best-performing test case, the classifier’s precision is 91.6% accompanied by 1% pseudo-overfitting, which is just slightly worse than the results achieved when training with the entire feature set: 92.6% precision and 3.7% pseudo-overfitting. This proves that the proposed FS algorithm is able to select the most important features in terms of their contribution to the prediction. Moreover, when reducing the number of features by 90% and 80%, the classifier’s precision increases by 1.5% and 2.7%, respectively. At first thought, this phenomenon might be unexpected, as one would think that the loss of information due to the reduction of the input will definitely lead to performance degradation. However, in a recent study, it has been proven that—even linear, correlation-based—FS indeed can improve the performance of a classifier neural network model [54]. This might be possible as FS also reduces the noise in the input data, which helps the model to generalize better.

A general observation is that *f* has a stronger impact on the outcome than final_prec. When training with a very small *f*, the pruning is performed in such slow steps, that the desired number of features cannot be reached within the maximum iteration limit. On the other hand, if it is too large, the weights are removed sooner than their value would stabilize, which leads to an improper selection strategy. Changing the final_prec does not evoke significant differences: with the fixed value of f=0.2, the results of the test cases remain close to each other even when choosing vastly different values for final_prec—see the test cases for selecting the top 5% of the original features. Nevertheless, f=0.2 and final_prec=0.75 seems to be an advantageous hyperparameter combination for selecting the top 5% of the features, and as we have seen, the changing of the final_prec does not have a significant effect on the outcome, further experiments are carried out using this setting.

### 5.2. Reproductibility

**Figure 11:**
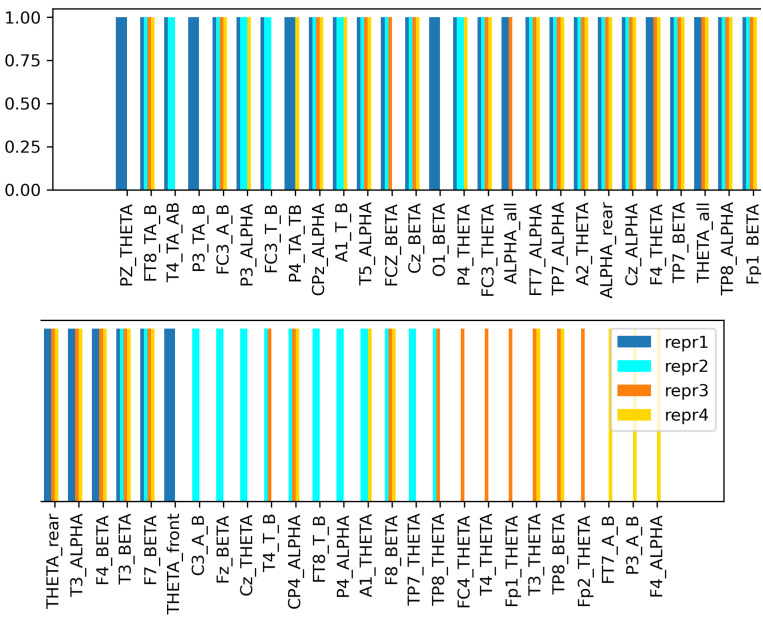
The selected features in 4 different sessions with the same hyperparameter settings: f=0.2, final_prec=0.75, des_feat_num=10%. Each color corresponds to a session, thus, features that are represented with more than one color, were selected into the final subset multiple times. This implies that these features have the highest predictive power.

The EEG cap that provides the data has a quite dense electrode distribution, meaning that the signals measured on the adjacent electrodes may be similar. Therefore, the original feature set with PSD values for each electrode will likely contain redundant information and many correlating features. Due to the random initialization of the weights in the classifier network and the highly correlated input features, occasionally different features may be selected into the final subset. This will result in slightly different performances in different runs when the FS is performed with the exact same settings. However, the features themselves might be different, the difference between the comprehensive descriptive power of the generated feature subsets is negligible. This is proven by the results in Table 6, which shows the performance of the FS algorithm from 4 different sessions, using the same settings in each of them: f=0.2, final_prec=0.75, des_feat_num=10% of the original set. Similarly as before, with each obtained feature subset, the base network was trained 4 times, and the averaged results are presented in the table. Even though the feature subsets are not completely the same (Figure 11), the performances achieved in the different sessions are close to one another.

### 5.3. Comparison to Other FS Methods

To get a thorough view of the proposed method’s efficiency, it is compared to the following conventional FS methods: PCA, random selection, Chi-squared est, MI analysis and RFE, that were introduced in Section 2.4.1. The top 5%, 10%, and 20% features of the original feature set have been selected using the aforementioned methods. In the case of PCA, the projection was not completed. Instead, the des_feat_num number of features that mostly contributed to the first principal component has been selected, and the base network is trained with them. The averaged results from 4 runs are summarized in Table 7. In all three scenarios, the proposed method outperforms all the examined conventional FS methods.The efficiency of the method is the most visible when reducing the initial feature set by 95%: it achieves more than 24% higher precision compared to PCA and even 3.2% higher precision compared to the best performing conventional FS method, the Chi-squared test.

**Figure 12:**
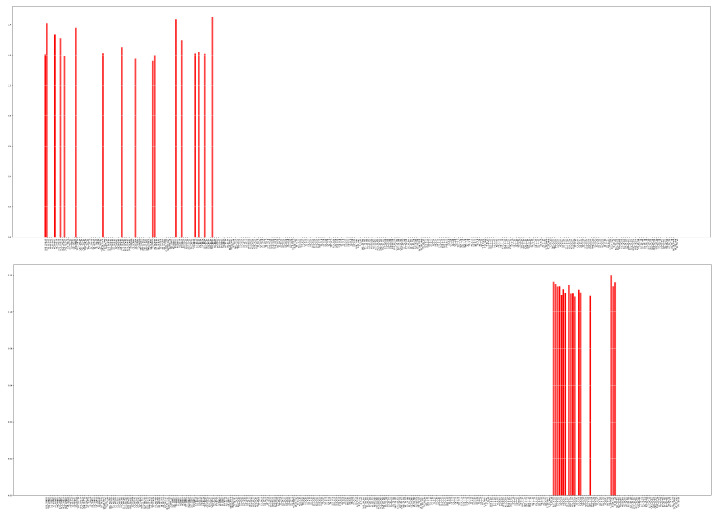
Selected TOP 5% features by the proposed algorithm (**up**) and by PCA (**bottom**).

Figure 12 shows the chosen features by the proposed method and PCA with their final scores. The selected features are highly dissimilar in the case of the two FS methods: while the proposed method mostly selects the sole α, θ, β PSDs, PCA assigns higher scores to the $\frac{\alpha }{\alpha +\theta }$ feature, measured on different electrodes.

## 6. Discussion

The proposed FS algorithm proves its efficiency by being able to reduce the original feature set even by 95% without major degradation in the performance: using the best performing hyperparameter setting, the classifier’s precision drops only by 1%. When moderately reducing the initial feature set, the proposed FS algorithm is able to reduce the noise and extract vital information. This is revealed by the results when selecting the top 10% and top 20% of the initial features, the classifier’s precision increases by 1.5% and 2.7%, respectively. Furthermore, it achieves better results on the given problem than the examined conventional FS methods.

The features selected by the proposed FS method also appear in other studies as best indicators of driver fatigue among other EEG features. In [55], they examined the relationship between reaction ability, physiological signals, and driving fatigue, and concluded that among the frequency domain features β-PSD has the greatest correlation with the reaction time based on Grey correlation analysis. This is due to the fact, that β waves appear in case of excitement or alertness. The experiments in study [56] show that the effect of the mutual addition of α, β and θ waves are more satisfactory compared to when these waves are used alone. Similarly to this conclusion, it can be seen that the proposed method always selects these α-PSD, β-PSD and θ-PSD based features together into the final subset, regardless of the different settings or runs (Figure 11). Lastly, the credibility of the θ-PSD’s presence in the final subsets is proven by the fact that high θ activity refers to the microsleep state [57], which indicates high-level drowsiness and sleep-onset state [58].

One drawback of the introduced solution is that the feature scoring relies on the weights in the classifier network, therefore in its current state, the FS is highly sensitive to the network’s random initialization; the results may slightly vary in different runs.

## 7. Conclusions

The stated goal—the development of a FS algorithm that can be later utilized for the development of a reliable drivers’ drowsiness detector—has been successfully achieved. Inspired by an idea introduced in a SOTA paper, we have designed an embedded FS method that exploits the neural networks’ working principle for feature scoring: the classifier network is supplemented with a an FPL that has the same size as the number of input features, is in a one-to-one relationship with them and the magnitude of its weights represent the importance of the corresponding features. In order to find the desired number of features with the best predictive power, the FPL was pruned iteratively during the classifiers’ training.

As EEG measures the electrical activities in the brain, and has been proven to be one of the best indicators of drowsiness, we have used this data source in this study. The initial feature set has been constructed from frequency-based features extracted from a public data set that contains raw EEG data recorded during sustained-attention driving tasks.

Using our FS algorithm, we were able to reduce the initial feature set by 95% without a significant deterioration in the drowsiness detection model’s precision. Additionally, we have showed its feature purification capabilities compared to linear projection. With this outstanding result, the proposed algorithm outperforms all of the tested conventional FS methods: random selection, PCA, RFE, Chi-squared test, and mutual information analysis.

Further plans include discovering the stabilization opportunities for the method and the comparison of its performance to a widely used nonlinear FS method. In addition, we also plan to examine its generalization ability over different drowsiness indicator features. Our final goal is to incorporate the proposed FS algorithm into the development process of our in-house drowsiness detector system.

## Figures and Tables

**Table 1:** Comparison of the FS method types [44] extended with complexity.

Type	Pros	Cons	Train-Time Complexity
Filter	Standalone tool Reduced risk of overfitting Fastest to calculate	Lack of feature dependencies No interaction with model	$\chi^{2}$ test: O(n·log(n)) Mutual Information Analysis: O(n·log(n))
Wrapper	Interacts with model Higher performance levels	High risk of overfitting Slowest construction Results are classifier dependent	Recursive feature elimination: (decision tree) O(n·log(n)·d·log(d))
Embedded	Integrated into model Highest performance levels	Middle-speed construction Results are classifier dependent	Stepwise Weight Pruning Algorithm: (neural network) O(*d*) + O(model)/per sample

**Table 2:** Thresholds for the feature pruning criteria.

*final_prec*	*max_epochs*	*max_psovft*
[0.6, 0.95]	20	0.05

**Table 3:** Results of predicting the drowsiness labels using different neural network architectures.

Name	Network Architecture	${\mathrm{prec}}_{\mathrm{avg}}$	${\mathrm{psovft}}_{\mathrm{avg}}$	${\mathrm{prec}}_{\mathrm{std}}$	${\mathrm{psovft}}_{\mathrm{std}}$
arch1	64; 32; 16; 2	0.893	0.029	0.004	0.0015
arch2	64; do; 32; do; 16; do; 2	0.881	0.016	0.0043	0.009
arch3	128; 64; 32; 2	0.907	0.032	0.0042	0.0042
arch4	128; do; 64; do; 32; do; 2	0.899	0.016	**0.0015**	**0.0013**
arch5	**256; 128; 64; 2**	**0.926**	0.037	0.042	0.004
arch6	256; do; 128; do; 64; do; 2	0.902	**0.014**	0.004	0.009

**Table 4:** Results achieved by the classifier when training with the original feature subset.

All Features (#330)	
***prec***	***psovft***
0.926	0.037

**Table 5:** Results of different sized feature subsets generated with the proposed FS method.

TOP 20% (#66)				TOP 10% (#33)				TOP 5% (#17)			
**Results**		**Hyperparameters**		**Results**		**Hyperparameters**		**Results**		**Hyperparameters**	
***prec***	***psovft***	***f***	***final_prec***	***prec***	***psovft***	***f***	***final_prec***	***prec***	***psovft***	***f***	***final_prec***
**0.953**	**0.033**	**0.2**	**0.95**	**0.941**	**0.028**	**0.2**	**0.7**	**0.916**	**0.01**	**0.2**	**0.75**
0.948	0.039	0.3	0.95	0.935	0.027	0.2	0.65	0.906	0.022	0.2	0.7
0.946	0.031	0.3	0.9	0.931	0.039	0.2	0.9	0.901	0.016	0.3	0.75
0.916	0.036	0.3	0.75	0.887	0.017	0.4	0.9	0.795	0.002	0.3	0.95

**Table 6:** Results of 4 different sessions with the same hyperparameter settings: f=0.2, final_prec=0.75, des_feat_num=10%.

Experiment	*prec*	*psovft*
repr1	0.924	0.036
repr2	0.932	0.025
repr3	0.938	0.025
repr4	0.927	0.027

**Table 7:** Classification performances when training with the subsets of the TOP 20%, 10%, and 5% of the original features generated using different FS methods. The indicated metrics—Accuracy (A), Precision (P), Recall (R) and F1-score (F1)—are calculated using macro averaging.

	TOP 20% (#66)				TOP 10% (#33)				TOP 5% (#17)			
**FS Method**	**A**	**P**	**R**	**F1**	**A**	**P**	**R**	**F1**	**A**	**P**	**R**	**F1**
random	0.523	0.648	0.571	0.607	0.55	0.539	0.548	0.55	0.51	0.504	0.51	0.48
PCA	0.887	0.886	0. 89	0.888	0.739	0.75	0.739	0.745	0.67	0.673	0.672	0.672
Chi	0.929	0.928	0.93	0.929	0.911	0.912	0.914	0.913	0.885	0.884	0.887	0.886
MI	0.925	0.926	0.928	0.927	0.908	0.908	0.911	0.91	0.842	0.85	0.841	0.846
RFE	0.911	0.91	0.912	0.911	0.88	0.88	0.88	0.88	0.82	0.831	0.822	0.826
proposed	**0.943**	**0.94**	**0.945**	**0.943**	**0.927**	**0.927**	**0.928**	**0.927**	**0.916**	**0.916**	**0.918**	**0.917**

## Data Availability

Not applicable.
